# Giant Cell Reparative Granuloma of Parotid Region Infiltrating the Zygomatic Bone: A case report

**DOI:** 10.1016/j.amsu.2020.06.014

**Published:** 2020-06-26

**Authors:** Suzan Sulaiman Alzaidi, Abdullah Mohammed Ghafouri, Sarah Abdulnasir Alayoubi, Yasser Abdullah Rhbeini

**Affiliations:** a Department of Otolaryngology and Head and Neck Surgery, King Fahad Armed Forces Hospital, Jeddah, Saudi Arabia; b College of Medicine, King Saud Bin Abdulaziz University for Health Sciences, Jeddah, Saudi Arabia; c Department of Maxillofacial Surgery, King Fahad Armed Forces Hospital, Jeddah, Saudi Arabia

**Keywords:** Giant cell reparative granuloma, Parotid region, Benign tumor, Surgical excision

## Abstract

**Introduction:**

Giant cell reparative granuloma is a rare, locally benign tumor with an aggressive behavior resembling malignant neoplasm, originating mainly from the mandible and maxilla; however, it can originate from other sites, such as zygomatic and temporal bones, with a young adult female predilection.

**Case presentation:**

A 28-year-old female presented to the Department of Otolaryngology and Head and Neck Surgery with a history of a slowly enlarging swelling over the left parotid region for four months. Physical examination revealed a non-erythematous, non-tender, firm mass with no overlying skin changes. Fine needle aspiration cytology of the lesion revealed a multinucleated giant cell-rich tumor and the histopathological examination of an incisional biopsy from the mass confirmed giant cell reparative granuloma. Therefore, a total surgical excision of the mass with curating of the zygomatic and temporal bones was performed with uneventful postoperative course and regular follow ups for one year.

**Conclusion:**

A growing mass in the parotid region in a young adult female with no history of trauma should raise the suspicion of giant cell reparative granuloma. Histopathological examination is the definitive tool for diagnosis, and surgical excision is the treatment modality of choice in such cases.

## Highlights

• Similar GCRG case has been only reported once in the literature.
• This is the second case reporting a GCRG.
• Young females with no history of trauma should raise suspicion of GCRG.
• We must give more thoughts into etiologies causing such reported cases.

## 1 Introduction

Giant Cell Reparative Granuloma (GCRG) was first described by Jaffe in 1953 as a rare, benign, locally aggressive tumor of the bone, originating mainly from the mandible and maxilla [1]. GCRG usually results from an inflammatory response to intraosseous hemorrhage following traumas; however, it may evolve spontaneously without a history of trauma [1,2] while affecting mainly children and young female adults [3]. As a result of the associated symptoms, as well as, the radiological findings of such cases being non-specific, the definitive diagnosis relies mainly on a histopathological examination of a biopsy taken from the mass. Although GCRG causes destruction and erosions of the adjacent bones and tissues, it carries a good prognosis after surgical excision with a low recurrence rate. We present a full clinical picture of a case with GCRG of the zygomatic bone along with the investigations conducted for reaching the definitive diagnosis, in addition to the way of management and postoperative outcome. To the best of our knowledge, there has been only one similarly reported case in the literature of a GCRG of a parotid region infiltrating the zygomatic bone [4]. This work has been reported in line with SCARE criteria [5].

## 2 Case Presentation

A 28-year-old medically free female presented to our clinic in the Department of Otolaryngology and Head and Neck Surgery with a history of a slowly enlarging swelling over the left parotid region for a duration of four months. On further questioning, there was no history of facial trauma or injury, family history nor smoking. The patient has not been on any drugs with no past psychological history and no genetic investigation was carried as it was irrelevant. Furthermore, the swelling was not related to any meal intake nor feeding problems and there were no constitutional symptoms reported. Upon physical examination, a 4 × 3 cm well-defined, non-erythematous, non-tender, non-fluctuant, firm swelling over the left parotid region with no overlying skin changes was seen. Moreover, no findings were noted on bimanual and ENT examinations and there were no palpable cervical lymph nodes. Therefore, a decision to take a Fine Needle Aspiration Cytology (FNAC) of the mass was made revealing the presence of multinucleated giant cells-rich tumor of uncertain malignant potential (Milan system for reporting salivary gland cytopathology category 4b).

**Fig. 1:**
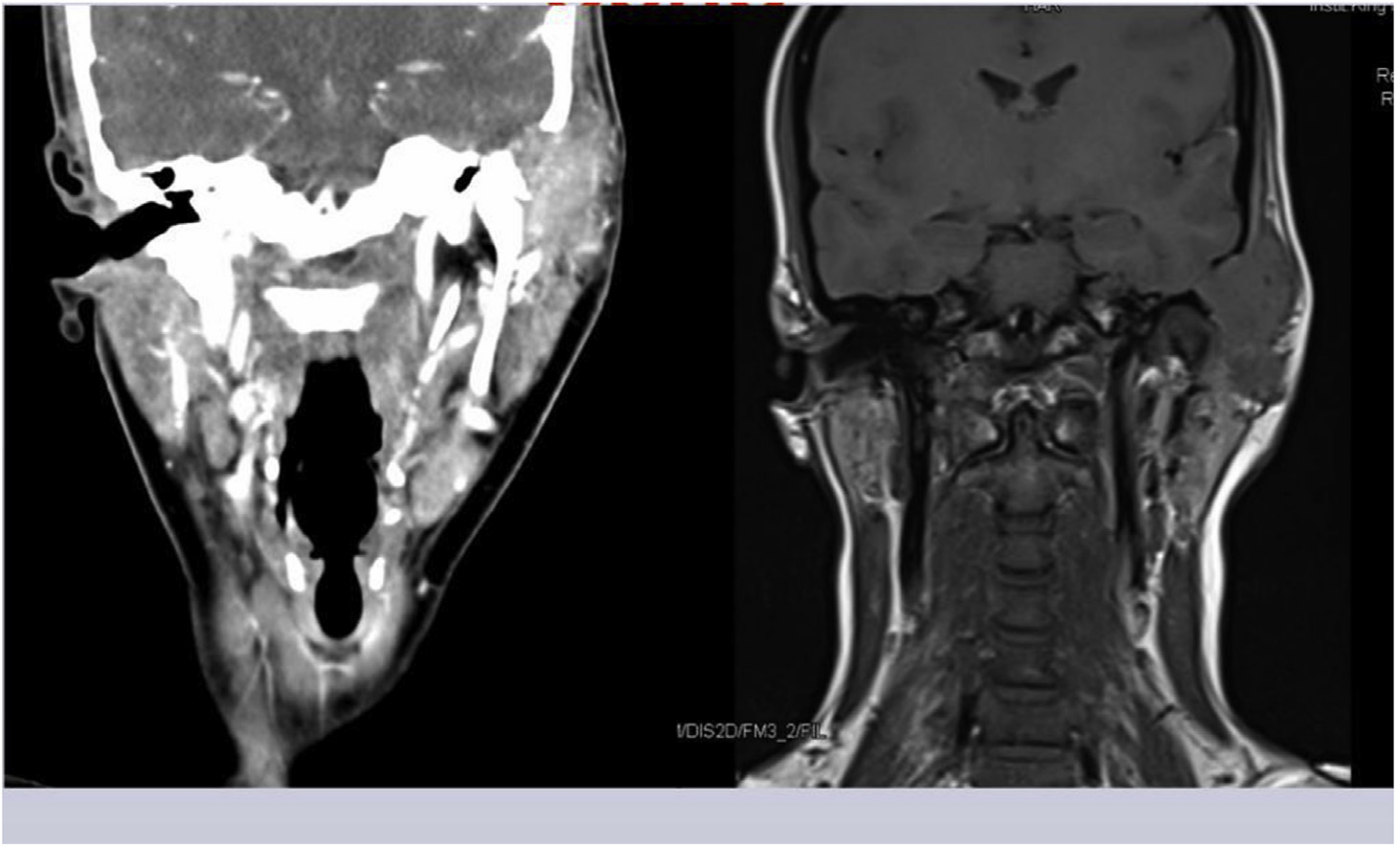
A Contrast-Enhanced Computed Tomography (CECT) displaying the mass with infiltration to the superficial lobe of the left parotid gland. Erosion of the left glenoid fossa, left temporal and zygomatic bones is seen.

Regarding radiological investigations, a Contrast-Enhanced Computed Tomography (CECT) scan displayed an ill-defined mass that measured 3.2 × 2.0 cm and infiltrated the superficial lobe of the left parotid gland. In addition, erosion of the left glenoid fossa, left temporal and zygomatic bones was noted with an exertion of a mass effect over the external auditory canal. Even though the mass was extended intracranially with bulging impression on the temporal fossa (Fig. 1) resulting in a compressed brain tissue, an intact dura was noted. Regarding magnetic resonance imaging, a Gadolinium-Enhanced Magnetic Resonance Imaging (MRI) of the brain and neck soft tissues was consistent with the CECT findings. However, the mass was seen to abut the meninges at the lateral aspect of the left temporal lobe in the left middle cranial fossa. To confirm the diagnosis, an incisional biopsy was taken from the mass under general anesthesia and sent for a histopathological examination, which whereby confirmed the presence of GCRG. The case was discussed in the tumor board and a decision to perform a total surgical excision of the mass in collaboration with neurosurgeons was made.

The procedure was decided and was conducted by one otolaryngology, head and neck oncology and reconstruction surgeon of 10 years' experience and a second otolaryngology and head and neck surgeon with 7 years’ experience. Intraoperatively, a pre-auricular incision was made through modified Blair incision with extension superiorly to the hairline margin in which a posterior flap was then raised at the level of the sternocleidomastoid fascia. Afterwards, the greater auricular nerve branches and the external jugular vein were identified and preserved, and the plane was developed where a hard mass was found being attached closely to branches of the facial nerve. Furthermore, retrograde dissection was made medial to lateral while the facial nerve was monitored and observed carefully using a facial nerve monitor (NIM 4 ports). The mass was then found deeply attached to the brain dura. At this point, the temporal bone was visualized, and a neurosurgeon joined to facilitate the need for further intervention. However, after careful evaluation only small parts of the temporal bone was eroded along with part of the zygomatic bone while the dura was intact. Tachosil was then used to seal the exposed dura and testing for cerebrospinal fluid showed no leakage present. Regarding the eroded bones, they were curetted and smoothened using a Volkmann Bone Curette with a periosteal elevator. Complete harvesting of mass was made and all facial Mercer tested positive with no reconstruction needed.

**Fig. 2:**
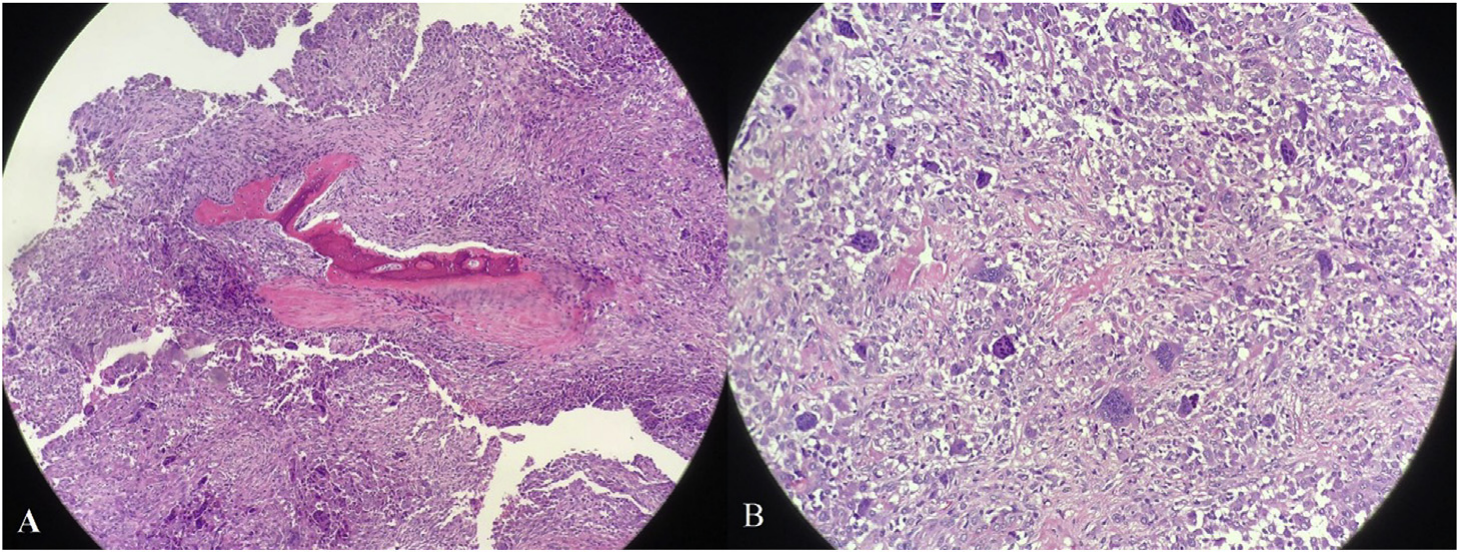
Histopathology slides showing A) irregular fragment of giant cell lesions and B) Several fragments of cortical bone with fragments of giant cells lesion attached to the bony fragment.

Regarding her postoperative course, the patient was taken to the surgical ward in a stable condition after placement of a drain with painkiller management regime of paracetamol, PRN oral narcotics as well as prophylactic antibiotic (co-amoxicillin) and demonstrated an uneventful and stable course; therefore, the drain was removed on the second day with minimum output. Additionally, the facial nerve was reassessed and was intact with no evidence of CSF leak on further examination. On the third postoperative day, the patient was discharged home in a good stable condition. Regarding the histopathological examination of the mass excised, it was 3.5x2.5 × 1 cm in size, infiltrating parotid tissue, zygomatic and temporal bones, muscle and fat without lymph nodes involvement, atypia, necrosis or increased mitotic activity of the cells. The final histopathology report demonstrated the presence of a mixture of multinucleated giant cells, osteoclast-like with more than 20 nuclei per cell. In addition, the stromal cells were found to be oval, spindle and round while satellite lesions were seen away from the main mass as a sign of infiltration of bony and fat tissues. Furthermore, thick walled vessels with endarteritis obliterans were seen at the periphery of the lesion (Fig. 2). Meanwhile, in regard to the parotid region lymph node, a reactive lymphoid hyperplasia with no metastatic lesion was seen and the left zygomatic bone fragment showed presence of the tumor present as an extension.

Accordingly, the oncology tumor board planned for follow-up only without any further interventions. Follow up was initially weekly post operatively for the first month, then monthly for the next 3 months. Afterwards, it became every 3 months for the following 6 months then every 6 months for 2 years, first surveillance scan was done 3 months postoperatively in form of an MRI and it was negative for recurrences followed by another MRI on a yearly basis for 2 years with future surveillance plan of having one MRI per year for the next 5 years.

## 3 Discussion

In 1953, Jaffe first described GCRG as fibro-osseous dysplasia, located mainly in the mandible and maxilla and to a lesser extent in the temporal, zygomatic, ethmoid, sphenoid and hand bones [1,6,7]. Despite the fact that the exact etiology remains unknown, it is suggested that GCRG follows an inflammatory reaction to intraosseous hemorrhage due to trauma which our case lacked. Nonetheless, GCRG can mimic other malignant neoplasm in having aggressive behavior, and tends to spread locally; therefore, it may involve the surrounding tissues, as well as nasal, temporal, and parietal bones. Histologically, GCGR consists of a mixture of multinucleated giant cells and fibroblasts with interspersed areas of hemorrhages. The immunohistochemical testing of GCRG is positive for vimentin, CD 31, CD56, and CD68, and reveals the presence of high levels of cell cycle protein Ki-67, a marker indicating increased cell proliferation [8,9]. Regarding age of GCRG occurrence, it is reported in the literature to affect mainly children as an unknow etiology as well as young adult females due to hormonal dependence suggested by a flare-up and recurrence during pregnancy [3]. Regardless, it is important to know that such cases of parotid region swelling can have a differential diagnosis of GCRG, brown tumor of hypothyroidism, giant cell tumor, fibrous dysplasia, aneurysmal bone cyst and parotid small cell carcinoma. Therefore, careful clinical, biochemical, and histopathological examination should be performed for reaching the definitive diagnosis and establishing a suitable treatment and follow-up plan. In comparison, GCRG can be differentiated from brown tumor of hyperparathyroidism, as the latter is associated with high levels of parathyroid hormone (PTH), alkaline phosphatase and serum calcium, in addition to a low level of serum phosphate. On the other hand, GCRG can be differentiated solely histopathologically from giant cell tumor (GCT), parotid small cell carcinoma and aneurysmal bone cyst. Despite the histological resemblances, GCT has larger giant cells with more nuclei compared to GCRG. In parotid small cell carcinoma, tumor cells show diffuse growth with necrosis in addition to the presence of several residual ducts and glands [10]. With regards to aneurysmal bone cyst, tumor mass contains sinusoidal blood spaces while fibrous dysplasia contains a scanty number of giant cells which merges with the cortical bone and usually ceases with maturity. Regarding the treatment modality of choice, total surgical excision with curettage is the preferred approach; however, other non-surgical treatment modalities are present such as intralesional corticosteroid injection and subcutaneous alpha-interferon injections but are less preferred [11]. Although GCRG has an aggressive behavior and tends to spread locally, it carries a good prognosis with a low incidence of recurrence [12]. Lastly, regular follow-up visits with radiological examination in parotid swellings are recommended for exclusion of any recurrence. Similar to our case, the only case to be reported in the literature with the same pattern is that of a 30-year-old female with no history of trauma and a slowly progressive swelling of the parotid region [4]. The patient was satisfied with the outcome and how the treatment plan was carried as well as the follow up afterwards.

## 4 Conclusion

GCRG occurs more frequently in children and young adult female. However, this is the second reported case in the literature to be reported arising from the parotid region. As more cases likely to be present, it is important to have such a rare pathology in mind in young adult females with no history of trauma presented with a slowly progressive swelling over the parotid region. Surgery is the mainstay of treatment for such cases.

## Ethical approval

Written informed consent was obtained from the patient for publishing this report with the included radiological and histopathological images.

## Funding

No funding was received.

## Contribution

All included authors contributed in writing, proofreading and referencing of this case report.

## Provenance and peer review

Not commissioned, externally peer reviewed.

## Declaration of competing interest

The authors declare no conflict of interest.
